# Comparison of Alignment Efficiency, Arch Dimensional Changes, and Passive Extraction Space Closure Between Self‐Ligating and Conventional Brackets in Extraction Crowding Patients: A Prospective Randomized Clinical Trial

**DOI:** 10.1155/ijod/4061065

**Published:** 2026-08-13

**Authors:** Marium Jamil, Fareeha Bokhari, Ayesha Hammad, Hassaan Saeed, Maliha Mumtaz, Muhammad Abdullah Kamran, Ali Alqerban, Umar Hussain, Ferdous Bukhary, Asif Rehman

**Affiliations:** ^1^ University College of Dentistry, University of Lahore, Lahore, Pakistan, uol.edu.pk; ^2^ Locum Specialist Consultant Orthodontist, Russels Hall Hospital, Dudley, UK; ^3^ Bashir Institute of Health Sciences, Islamabad, Pakistan; ^4^ Orthodontics Division, Department of Pediatric Dentistry and Orthodontic Sciences College of Dentistry, King Khalid University, Abha, Saudi Arabia, kku.edu.sa; ^5^ Department of Pediatric Dentistry, College of Dentistry, Prince Sattam Bin Abdulaziz University, Al-Kharj 11942, Saudi Arabia, psau.edu.sa; ^6^ Saidu College of Dentistry, Swat, Minogra, Pakistan; ^7^ Department of Preventive Dental Sciences, College of Dentistry, Dar Al Uloom University, Riyadh, Saudi Arabia, dau.edu.sa; ^8^ Bath Spa University, Bath, UK, bathspa.ac.uk

**Keywords:** alignment, intercanine width, intermolar width, passive extraction space, self-ligating brackets

## Abstract

**Objective:**

To compare the alignment efficiency, arch dimensional changes, and passive extraction space closure between between self‐ligating brackets (SLBs) and conventional brackets (CBs).

**Materials and Methods:**

This randomized controlled trial on 70 participants (13–30 years) with moderate to severe crowding, skeletal Class I or mild to moderate Class II malocclusions, all requiring premolar extractions, who were randomly assigned to CB (*n* = 35) or SLB (*n* = 35) groups. Baseline age, gender, crowding, and malocclusion type were recorded. Standardized 0.022‐inch MBT brackets were bonded, with alignment started using 0.014‐inch nickel‐titanium (NiTi) archwires and monthly follow‐ups. Outcomes—Little’s Irregularity Index, intercanine and intermolar widths, and passive extraction space closure—were measured on blinded casts at baseline, 3, 6, and 9 months using digital calipers, with no active space‐closing auxiliaries. Data were analyzed using linear mixed‐effects models with repeated measures and covariates; regression coefficients with 95% CIs were reported, and *p* ≤ 0.05 was considered significant.

**Results:**

Over the 9‐month observation period, both groups showed substantial improvement in alignment. SLBs demonstrated faster early alignment, with greater reductions in Little’s Irregularity Index at 3 months in both arches (maxilla: β = −0.91, *p*  < 0.001; mandible: β = −0.60, *p* = 0.009), and in the maxilla at 6 months (β = −0.66, *p* = 0.005). Intercanine width increased in both groups, but expansion was consistently smaller with SLBs (β = −1.36 to −1.69 mm; all *p*  < 0.001). In contrast, intermolar width was greater with SLBs throughout follow‐up in both arches (maxilla: β = 2.34–2.77 mm; mandible: β = 2.06–2.34 mm; all *p*  < 0.001). Passive extraction space closure decreased significantly over time in both groups, with no significant effect of bracket type.

**Conclusion:**

SLBs showed smaller increases in intercanine width, greater intermolar expansion, and faster early alignment. However, passive space closure was similar between groups. Differences were modest and may have limited clinical significance.

**Trial Registration**: Retrospectively registered at ClinicalTrials.gov (NCT07503314)

## 1. Introduction

The goal of orthodontic treatment is to place teeth in esthetically pleasing, functionally efficient, and stable position [1]. Proper selection of the appliance is necessary to achieve the required precise control of tooth movement, preserve arch form, and reduce treatment duration [2]. Short treatment duration can minimize adverse effects such as pain, enamel demineralization, white spot lesions, and difficulties with oral hygiene [3, 4].

During comprehensive fixed appliances treatment, the control over arch dimensions, particularly intercanine and intermolar widths, is critical not only for esthetics but also for long‐term stability [5]. Wider posterior arches are generally considered more attractive, may reduce the need for extractions, and provide a favorable foundation for functional occlusion [6]. Maintenance of intercanine width is also an important predictor of alignment stability [7].

Orthodontic treatment typically employs either conventional brackets (CBs), which use elastic or wire ligatures to secure the archwire inside the brackets, or self‐ligating brackets (SLBs), which feature a built‐in clip or sliding gate mechanism. SLBs offer key advantages by eliminating the need for certain materials such as elastomeric modules [8]. This results in several benefits, including reduced risk of cross‐contamination from elastic ligatures, consistent full engagement without force loss from elastomeric relaxation, potentially lower enamel decalcification due to fewer plaque‐retentive areas, theoretically less friction during sliding mechanics, and the use of lighter forces that may minimize side effects like root resorption and pain [9].

SLBs were introduced with the idea that eliminating ligature ties reduces friction between the archwire and the bracket slot, creating a relatively frictionless environment [10]. This design is intended to facilitate smoother sliding mechanics, allowing teeth to move more freely along the archwires. As a result, alignment can progress faster, especially during the early stages of treatment, while lighter forces may improve patient comfort [11]. SLBs may also exert a significant influence on arch dimensions. By minimizing friction and permitting more unrestricted tooth movement, these appliances may help maintain intercanine width, thereby reducing the risk of constriction or severe expansion and hence reduce post‐treatment relapse. It is also claimed that the posterior segments can undergo more controlled lateral expansion, resulting in increased intermolar width. Such modifications can enhance both esthetic wider arches and high occlusal stability, while potentially decreasing the need for tooth extractions or interproximal reduction [12]. In extraction cases, passive space closure is a key phase of orthodontic treatment. The reduced friction associated with SLBs may facilitate more efficient and predictable mesial movement of posterior teeth without the use of active auxiliaries, thereby promoting controlled space closure and potentially shortening the overall treatment duration [13].

Despite the widespread clinical use of SLBs, the advantages attributed to these systems over CBS, particularly in relation to alignment efficiency, transverse arch changes, and space management, remain uncertain. The available evidence is inconsistent and often dependent on clinical context. Many earlier studies have primarily examined nonextraction cases, short observation periods, or single outcome measures, which limits the applicability of their findings to routine orthodontic practice where extractions, space closure, and transverse changes commonly occur together. The influence of bracket design on intercanine width, intermolar width, and passive extraction space closure therefore remains insufficiently understood, particularly when important variables such as malocclusion type, baseline crowding, and treatment mechanics are controlled [3, 14–16]. Previous network meta‐analyses included highly heterogeneous studies with different malocclusions, extraction and nonextraction cases, treatment mechanics, bracket systems, and follow‐up periods, which may mask subgroup‐specific effects [17]. In contrast, the present study focused exclusively on extraction crowding cases managed with a standardized protocol, fixed review intervals, and uniform archwire progression. A key novel aspect was the prospective measurement of passive extraction space closure during the leveling phase without active auxiliaries, allowing the independent influence of the bracket design to be assessed. Repeated assessments at baseline, 3, 6, and 9 months also enabled evaluation of the pattern and timing of early alignment and transverse arch changes, providing more clinically relevant evidence for extraction‐based orthodontic treatment.

The present study was undertaken to provide a longitudinal comparison of SLBs and CBs in a well matched extraction cohort, with concurrent evaluation of alignment efficiency, transverse dimensional changes, and passive space closure within the same population. By assessing these clinically relevant outcomes at multiple time points using adjusted regression analyses, this study aims to determine whether the proposed biomechanical advantages of SLBs translate into meaningful and sustained differences in treatment response, thereby guiding evidence based bracket selection in contemporary orthodontic practice.

## 2. Methodology

### 2.1. Study Design and Trial Registration

This single‐blind, parallel‐arm, prospective randomized controlled trial was conducted at XXX from February 1, 2024, to June 25, 2025, in accordance with CONSORT guidelines. Ethical approval was obtained from the institutional review committee of the University of Lahore prior to participant recruitment (Ref No: UCD/ERCA/20/11a). The trial was retrospectively registered at ClinicalTrials.gov (NCT07503314). Final statistical analyses were conducted after registration.

### 2.2. Sample Size Calculation

Sample size was calculated using a two‐sample comparison of means with a significance level of 5% (α = 0.05) and 80% power. Based on post‐treatment maxillary intermolar width values reported by Pandis et al. [18], with a mean of 44.64 mm (SD 2.74) in the CB group and 46.25 mm (SD 1.72) in the self‐ligating bracket group, the expected mean difference was 1.61 mm with a pooled standard deviation of 2.29 mm. This yielded a required sample of 32 participants per group (64 total). To account for possible attrition, the final sample size was increased to 70 participants (35 per group). As reliable extraction case data for alignment outcomes were limited at the time of protocol development, intermolar width was used as the reference variable for sample size estimation.

### 2.3. Patient Recruitment and Eligibility Criteria

Non‐probability consecutive sampling was used to recruit 90 eligible participants who met the inclusion criteria from the Department of Orthodontics, University Dental Hospital, University College of Medicine and Dentistry, University of Lahore.

The inclusion criteria were patients aged 13–30 years of either gender, presenting with moderate (4–8 mm) or severe (>8 mm) dental crowding, skeletal Class I (ANB = 0–2^0^) or mild to moderate skeletal Class II malocclusions (ANB = 3–5^0^), and normal or vertical growth patterns (FMA = 22–28^0^). Only patients with good oral hygiene, defined as a plaque index ≤ 1 according to Silness and Löe, were included. As one of the study outcomes was passive space closure, only first premolar extraction cases were selected.

Patients were excluded if they had a history of previous orthodontic treatment, requiring asymmetric extractions, systemic diseases (e.g., diabetes or osteoporosis), or were taking medications known to affect bone metabolism or healing (such as corticosteroids or bisphosphonates). Individuals with cleft lip and palate or other craniofacial anomalies and those with congenital absence of teeth other than third molars were also excluded. Low‐angle (hypodivergent) cases were omitted to minimize biomechanical variability as differences in bone density and muscular strength in such patients may alter the rate and pattern of orthodontic tooth movement, thereby confounding intergroup comparisons.

### 2.4. Randomization and Allocation Concealment

**Figure 1:**
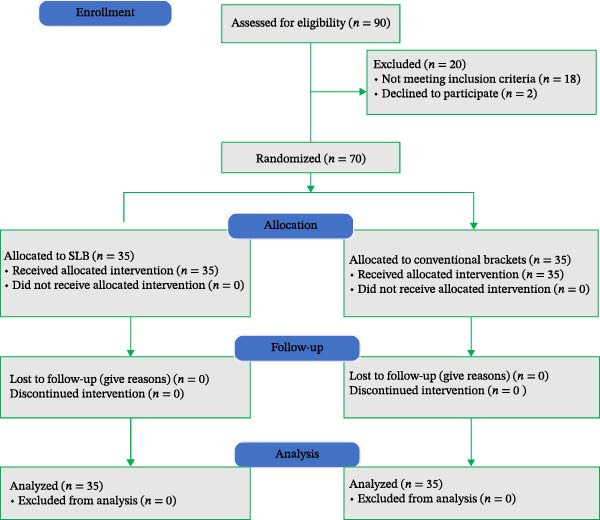
CONSORT flowchart of participants through the trial.

A total of 90 patients scheduled for fixed appliance treatment were identified from the appointment register and assessed for eligibility. Following a detailed clinical examination, 18 patients did not meet the inclusion criteria and were excluded. Two additional eligible patients were excluded because they relocated abroad before treatment could commence. The remaining 70 participants were enrolled and randomized. Randomization was performed using IBM SPSS Statistics. A simple randomization method was used to allocate the remaining 70 participants into two equal groups (1:1 ratio): Group 1 (SLBs) and Group 2 (CBs). The random allocation sequence was generated using computer‐produced random numbers by an independent researcher who was not involved in treatment delivery or outcome assessment. Allocation concealment was ensured using sequentially numbered, opaque, sealed envelopes containing group assignments. The envelopes were prepared by the independent researcher, stored in a secure, locked cabinet, and opened sequentially by the study coordinator only after completion of baseline records and confirmation of eligibility. Because of the nature of the interventions, blinding of the operator and participants was not feasible. However, outcome assessment and data analysis were performed under blinded conditions. The examiner responsible for model measurements was unaware of bracket type and group allocation. Brackets were masked on study models using beading wax (Figure 1).

### 2.5. Pretreatment Assessment

Baseline characteristics, including age, sex, amount of initial crowding, and malocclusion type were recorded for all participants. At baseline (*T*_0_), alginate impressions and intraoral photographs were obtained for all subjects. Dental casts were poured using Type III dental stone. First permanent molars in both arches were banded at the start of treatment. Following completion of the alignment phase, a transpalatal arch fabricated from 0.036‐inch stainless‐steel wire was inserted in the maxillary arch to reinforce anchorage during the subsequent working stage. Little’s Index of Irregularity, intercanine width, and intermolar width were measured on study casts using digital calipers (Series 600‐ Manual, Absolute Digimatic; Auto Motor Zuberhor, Hannover, Germany).

### 2.6. Interventions

In Group 1, Empower 2 brackets (0.022‐inch slot, MBT prescription; self‐ligating bracket American Orthodontics, Sheboygan, WI, USA) were bonded, whereas Group 2 was bonded with Equilibrium 2 brackets (0.022‐inch slot, MBT prescription; CBs, Dentaurum, Ispringen, Germany). The SLBs used in this study were interactive, remaining passive during the initial alignment and becoming active during the leveling phase. Brackets were bonded on the labial surfaces of teeth up to the second premolars in both arches using Transbond XT light‐cured adhesive (3M Unitek, Monrovia, CA, USA), following the manufacturer’s instructions and under standardized bonding conditions.

Alignment was started with a 0.014‐inch NiTi archwire (TruFlex, Ortho Technology Inc., Tampa, FL, USA) cinched at both distal ends. Passive stainless‐steel lacebacks (0.010‐inch wire) were placed between the canine and molar in each quadrant to control canine position during alignment and were not activated to produce canine retraction or space closure. Patients were reviewed at 4‐week intervals for activation. Data were collected at four time points: baseline (*T*_0_), 3 months (*T*_1_), 6 months (*T*_2_), and 9 months (*T*_3_). At each interval, impressions of both arches were obtained after blocking out the brackets with beading wax to facilitate cast removal and ensure concealment of bracket type for blinded model assessment. If alignment was completed before the 9‐month period, data collection was concluded at the visit corresponding to full alignment, and no further impressions were taken.

Throughout the observation period, overjet, overbite, molar relationship, and oral hygiene were routinely monitored to ensure uniform treatment progress. The standardized archwire sequence during the leveling and alignment phase included 0.014‐inch, 0.016‐inch, and 0.018‐inch round NiTi wires, followed by 0.016 × 0.022‐inch rectangular NiTi and 0.019 × 0.025‐inch rectangular stainless‐steel wires (TruForce, Ortho Technology Inc., Tampa, FL, USA). No elastic band or power chains were used for space closure during the leveling and alignment stage.

All treatments were performed by Fellowship of College of Physicians and Surgeons Pakistan (FCPS) orthodontic residents with a minimum of 2 years of clinical experience. Before trial initiation, all operators underwent calibration sessions to ensure consistency in bonding protocol, archwire progression, and ligation method across both groups. The operators were also given a written pamphlet about these procedures.

### 2.7. Outcome Measurement

All outcome measurements were recorded by a single calibrated examiner to ensure consistency and minimize measurement bias. The primary outcomes were Little’s Irregularity Index, used to assess alignment, and maxillary intermolar width, used to assess posterior transverse arch change. Secondary outcomes included intercanine width and passive extraction space closure.

Measurements were taken at baseline, 3, 6, and 9 months. Study casts of both the maxillary and mandibular arches were obtained at baseline (*T*_0_), 3 months (*T*_1_), 6 months (*T*_2_), and 9 months (*T*_3_). The degree of dental alignment was evaluated using Little’s Irregularity Index. Measurements were performed on all casts using a digital electronic caliper (Absolute Digimatic Series 600; Auto Motor Zubehör, Hannover, Germany) with an accuracy of 0.01 ± 0.02 mm. Calibration was performed prior to each measurement session using a standard reference gauge to maintain precision.

Passive space closure was assessed throughout the alignment phase. No active space‐closing auxiliaries, such as power chains or nickel–titanium (NiTi) closed‐coil springs, were used during this period to prevent additional biomechanical effects and isolate the influence of bracket design on tooth movement. Extraction space was measured from the nearest reproducible points on the crowns adjacent to the extraction site. Contact points were avoided due to potential distortion from rotations or tipping, and mesiodistal tooth widths were not used because displacement from the arch form could underestimate space dimensions.

**Figure 2:**
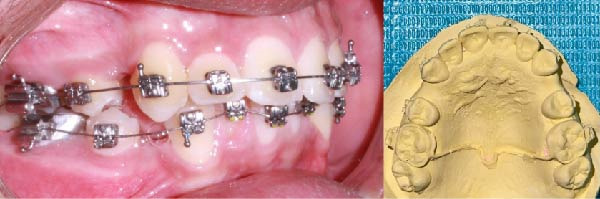
Clinical photograph of a participant in the self‐ligating bracket group during leveling and alignment after premolar extraction (left) and study cast used for outcome measurements (right).

Intercanine width was measured between the cusp tips of the canines, while intermolar width was measured between the mesial occlusal pits of the first molars as these landmarks provide higher impression fidelity than cusp tips (Figure 2).

All impressions were poured within 30 min using Type III dental stone, and casts were stored in a dry, temperature‐controlled environment until measurement. To assess intraexaminer reliability, 20 randomly selected casts (10 upper and 10 lower) were remeasured 2 weeks apart. Intraclass correlation coefficients (ICC) exceeded 0.90 for all parameters, confirming excellent measurement reproducibility.

### 2.8. Statistical Analysis

Data were analyzed using R. Descriptive statistics were calculated for all variables. Categorical variables (e.g., gender and malocclusion type) were summarized as frequencies and percentages, whereas continuous variables (e.g., age, Little’s Irregularity Index, intercanine width, intermolar width, and passive extraction space) were presented as means ± standard deviations or medians with interquartile ranges (IQR), according to data distribution. Normality of continuous variables was assessed using the Shapiro–Wilk test. Baseline characteristics were described for both groups without formal significance testing in accordance with CONSORT recommendations. The primary outcomes were Little’s Irregularity Index and maxillary intermolar width. Secondary outcomes included intercanine width and passive extraction space closure. Longitudinal changes in outcomes were analyzed using linear mixed‐effects models, with participant identifier included as a random intercept to account for repeated measurements within subjects. Fixed effects included treatment group, time, and the group × time interaction. Age, gender, and malocclusion type were entered as covariates. Regression coefficients (β) with 95% confidence intervals (CI) were reported. Estimated marginal means were derived for key between‐group comparisons over time. A two‐sided *p* value <0.05 was considered statistically significant. Model assumptions were assessed using residual‐versus‐fitted and normal Q–Q plots; no major deviations were observed.

## 3. Results

### 3.1. Model Diagnostic Assessment

**Figure 3:**
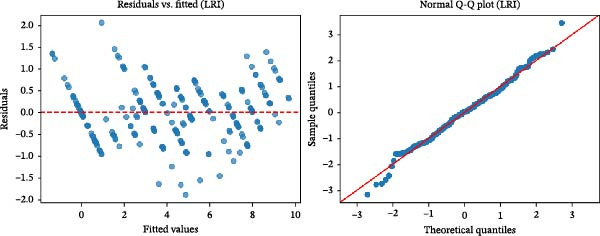
Diagnostic plot for your mixed‐effects model for Little’s Irregularity Index.

Diagnostic assessment of the mixed‐effects models demonstrated no major violations of assumptions. Residual plots showed acceptable homoscedasticity and approximate normality. For the LRI model, the ICC was 0.61, indicating substantial within‐subject correlation over repeated measurements (Figure 3).

### 3.2. Baseline Data

A total of 70 participants were enrolled, with 35 allocated to the CB group and 35 to the SLB group. The mean age was similar between groups (22.4 ± 4.94 years in the CB group and 21.9 ± 4.48 years in the SLB group). Females comprised 54.3% of the CB group and 62.9% of the SLB group. Class I malocclusion was present in 45.7% of the CB group and 51.4% of the SLB group, while the remainder had Class II malocclusion. Baseline demographic and clinical characteristics were similar in distribution across groups (Table 1).

**Table 1:** Baseline demographic and clinical characteristics of participants in the SLB and CB groups (*n* = 70).

Parameter	Characteristic	Conventional brackets (*n* = 35)	Self‐ligating brackets (*n* = 35)
Age (years)	Mean (SD)	22.4 (4.94)	21.9 (4.48)
Gender	Female	19 (54.3)	22 (62.9)
	Male	16 (45.7)	13 (37.1)
Malocclusion	Class I	16 (45.7)	18 (51.4)
	Class II	19 (54.3)	17 (48.6)
Maxilla, Median (IQR)	Little’s irregularity index at baseline	7.5 (6, 9)	8 (7, 9)
	Intercanine width at baseline (mm)	37 (33, 41.25)	37 (33, 41)
	Intermolar width at baseline (mm)	45 (43.5, 47)	44.5 (42, 45.5)
	Passive extraction space at baseline (mm)	4 (3.5, 5)	4 (3.25, 4.5)
Mandible, Median (IQR)	Little’s irregularity index at baseline	8 (7, 9)	8 (7, 8.5)
	Intercanine width at baseline (mm)	31 (27, 35.5)	31 (27, 35)
	Intermolar width at baseline (mm)	42 (40.5, 44)	42 (39, 43)
	Passive extraction space at baseline (mm)	4 (3.5, 4.5)	4 (3.25, 4.5)

### 3.3. Changes Over Time in Alignment, Arch Dimensions, and Passive Extraction Space Closure

Descriptive longitudinal summaries are shown in Table 2. In both groups, Little’s Irregularity Index progressively decreased from baseline to 9 months in the maxillary and mandibular arches, indicating substantial alignment improvement over time. Median values approached zero by 9 months in both groups, demonstrating near‐complete alignment at the end of the follow‐up. At the interim assessments (3 and 6 months), median irregularity scores were lower in the SLB group than in the CB group, suggesting faster early alignment with SLBs. Intercanine width increased over time in both arches, with consistently larger median values in the CB group at follow‐up, suggesting greater anterior transverse expansion with CBs. In contrast, the intermolar width was consistently greater in the SLB group at 3, 6, and 9 months in both arches, indicating a tendency toward greater posterior transverse expansion with SLBs. Passive extraction space measurements gradually decreased over time in both groups, reflecting spontaneous space closure during the alignment phase. However, median values remained broadly similar between groups throughout follow‐up, suggesting no clinically meaningful difference in passive space closure between bracket systems.

**Table 2:** Changes in alignment, arch widths, and passive extraction space closure at various time points in conventional and self‐ligating brackets in both arches.

Arch	Variable	Time point (months)	Conventional brackets (*n* = 35) Median (IQR)	Self‐ligating brackets (*n* = 35) Median (IQR)
Maxilla	Little’s irregularity index (mm)	3	6 (5, 6.5)	5 (4, 6)
		6	4 (3, 4)	3 (2.25, 4)
		9	0 (0, 0.5)	0 (0, 0)
	Intercanine width (mm)	3	39 (34, 43)	37 (33.25, 40.75)
		6	40 (36, 44.5)	38 (34.5, 41.75)
		9	40 (36, 44.5)	38 (34.5, 42.5)
	Intermolar width (mm)	3	44 (43.5, 47)	46.5 (44, 49)
		6	44 (43.75, 47.5)	46.5 (44.5, 49.5)
		9	44.5 (44, 48)	46.5 (44.5, 51)
	Passive extraction space closure (mm)	3	4 (3.5, 4.5)	4 (3, 4.5)
		6	4 (3.5, 4.5)	4 (3, 4)
		9	4 (3.5, 4.5)	4 (3, 4)
Mandible	Little’s irregularity index (mm)	3	6 (5, 6.5)	5 (4, 6)
		6	4 (3, 4)	3 (2.25, 4)
		9	0 (0, 1)	0 (0, 0)
	Intercanine width (mm)	3	33 (28, 37)	31 (27, 35)
		6	34 (30, 38.5)	32 (28, 36)
		9	34 (30, 38.5)	32 (28.75, 36)
	Intermolar width (mm)	3	41 (40.5, 44)	44 (41, 46)
		6	41 (41, 45)	44 (42, 47)
		9	42 (41.5, 45)	44 (42, 48)
	Passive extraction space closure (mm)	3	4 (3.5, 4.5)	4 (3, 4.5)
		6	4 (3.5, 4.5)	4 (3, 4)
		9	4 (3.5, 4.5)	3 (3, 4)

### 3.4. Mixed‐Effects Model Results

Linear mixed‐effects models adjusted for age, gender, and malocclusion type confirmed significant improvement in alignment over time in both arches. In the maxilla, the SLB group demonstrated significantly greater early reduction in Little’s Irregularity Index at 3 months (β = −0.91, 95% CI −1.37 to −0.46) and 6 months (β = −0.66, 95% CI −1.11 to −0.21), with no significant difference at 9 months. A similar early advantage for SLB was observed in the mandible at 3 months but not at later time points. For transverse dimensions, the intercanine width increased significantly over time, but the increase was consistently smaller in the SLB group in both arches. Conversely, the intermolar width increased significantly more in the SLB group at all follow‐up visits in both arches, supporting greater posterior arch expansion with SLBs. Passive extraction space closure decreased significantly over time in both arches; however, no significant treatment‐group effects or group‐by‐time interactions were observed, indicating similar rates of spontaneous space closure with conventional and SLBs. Age, gender, and malocclusion type were not significant predictors for most outcomes.

## 4. Discussion

This study compared CBs and SLBs in 70 patients with comparable baseline characteristics. Our findings indicate that SLB was more effective in maintaining intercanine width, achieving greater intermolar expansion, and producing a larger reduction in the irregularity index, whereas no significant differences were observed between the two bracket systems in passive space closure.

In the maxillary arch, SLBs demonstrated a significantly faster early reduction in Little’s Irregularity Index (LII, mm) compared with CBs. At 3 months, SLBs produced an additional reduction of 0.91 mm, corresponding to an estimated time gain of ~1.2 months (monthly alignment rate: 0.72 mm/month; 0.91 ÷ 0.72 ≈ 1.25 months). At 6 months, this advantage decreased to 0.66 mm, equivalent to a time gain of about 1 month. By 9 months, the difference between the bracket systems was no longer statistically significant, indicating convergence of alignment outcomes over time (Table 3). Overall, CBs required ~1–1.25 additional months to achieve comparable maxillary alignment with SLBs.

**Table 3:** Mixed‐effects regression analyses for alignment, arch width changes, and passive extraction space closure in maxillary and mandibular arches.

Jaw	Parameter	Predictor	Estimate (β) (95% CI)	*p*‐Value
Maxilla	Little’s irregularity index	Intercept	7.53 (6.36 to 8.70)	<0.001
		Group SLB	0.22 (−0.29 to 0.73)	0.405
		Time 3 months	−2.17 (−2.49 to −1.85)	<0.001
		Time 6 months	−4.34 (−4.66 to −4.02)	<0.001
		Time 9 months	−7.29 (−7.61 to −6.97)	<0.001
		Age (years)	0.02 (−0.03 to 0.07)	0.450
		Gender (male)	0.16 (−0.28 to 0.61)	0.477
		Malocclusion Class II	−0.34 (−0.78 to 0.10)	0.137
		Group SLB × Time 3 months	−0.91 (−1.37 to −0.46)	<0.001
		Group SLB × Time 6 months	−0.66 (−1.11 to −0.21)	0.005
		Group SLB × Time 9 months	−0.39 (−0.84 to 0.07)	0.096
	Intercanine width	Intercept	35.86 (30.78 to 40.93)	<0.001
		Group SLB	−0.37 (−2.32 to 1.57)	0.707
		Time 3 months	0.27 (−0.23 to 0.77)	0.290
		Time 6 months	1.27 (0.77 to 1.77)	<0.001
		Time 9 months	1.41 (0.91 to 1.92)	<0.001
		Age (years)	0.05 (−0.16 to 0.26)	0.655
		Gender (male)	1.77 (−0.18 to 3.73)	0.081
		Malocclusion Class II	−1.30 (−3.23 to 0.64)	0.193
		Group SLB × Time 3 months	−1.36 (−2.07 to −0.65)	<0.001
		Group SLB × Time 6 months	−1.61 (−2.32 to −0.90)	<0.001
		Group SLB × Time 9 months	−1.50 (−2.21 to −0.79)	<0.001
	Intermolar width	Intercept	46.45 (42.96 to 49.93)	<0.001
		Group SLB	−1.01 (−2.35 to 0.33)	0.143
		Time 3months	−0.17 (−0.55 to 0.21)	0.374
		Time 6months	0.13 (−0.25 to 0.51)	0.505
		Time 9months	0.39 (0.01 to 0.76)	0.046
		Age (years)	−0.06 (−0.20 to 0.08)	0.405
		Gender (male)	0.48 (−0.86 to 1.83)	0.483
		Malocclusion Class II	−0.39 (−1.72 to 0.94)	0.570
		Group SLB × 3months	2.34 (1.81 to 2.88)	<0.001
		Group SLB × 6months	2.54 (2.01 to 3.08)	<0.001
		Group SLB × 9months	2.77 (2.24 to 3.31)	<0.001
	Passive extraction space closure	Intercept	4.15 (3.32 to 4.98)	<0.001
		Group SLB	−0.13 (−0.46 to 0.20)	0.427
		Time 3 months	−0.09 (−0.19 to 0.03)	0.108
		Time 6 months	−0.19 (−0.29 to −0.08)	<0.001
		Time 9 months	−0.20 (0.01 to 0.36)	<0.001
		Age (years)	−0.01 (−0.04 to 0.02)	0.660
		Gender (male)	0.20 (−0.13 to 0.53)	0.226
		Malocclusion Class II	0.09 (−0.23 to 0.41)	0.575
		Group SLB × Time 3 months	−0.03 (−0.17 to 0.11)	0.704
		Group SLB × Time 6 months	−0.10 (−0.25 to 0.05)	0.185
		Group SLB × Time 9 months	−0.11 (−0.26 to 0.04)	0.130

Mandible	Little’s irregularity index	Intercept	7.50 (6.36 to 8.64)	<0.001
		Group SLB	−0.10 (−0.60 to 0.40)	0.694
		Time 3 months	−2.09 (−2.40 to −1.77)	<0.001
		Time 6 months	−4.29 (−4.60 to −3.97)	<0.001
		Time 9 months	−7.20 (−7.52 to −6.88)	<0.001
		Age (years)	0.02 (−0.03 to 0.07)	0.435
		Gender (male)	0.12 (−0.32 to 0.55)	0.596
		Malocclusion Class II	−0.36 (−0.79 to 0.07)	0.107
		Group SLB × Time 3 months	−0.60 (−1.05 to −0.15)	0.009
		Group SLB × Time 6 months	−0.37 (−0.82 to 0.08)	0.105
		Group SLB × Time 9 months	−0.10 (−0.55 to 0.35)	0.662
	Intercanine width (mm)	Intercept	29.88 (24.81 to 34.95)	<0.001
		Group SLB	−0.39 (−2.33 to 1.55)	0.694
		Time 3 months	0.24 (−0.26 to 0.75)	0.347
		Time 6 months	1.20 (0.70 to 1.71)	<0.001
		Time 9 months	1.39 (0.88 to 1.89)	<0.001
		Age (years)	0.04 (−0.16 to 0.25)	0.674
		Gender (male)	1.81 (−0.15 to 3.76)	0.074
		Malocclusion Class II	−1.28 (−3.21 to 0.65)	0.199
		Group SLB × Time 3 months	−1.39 (−2.10 to −0.67)	<0.001
		Group SLB × Time 6 months	−1.69 (−2.40 to −0.97)	<0.001
		Group SLB × Time 9 months	−1.53 (−2.24 to −0.82)	<0.001
	Intermolar width (mm)	Intercept	43.54 (40.05 to 47.04)	<0.001
		Group SLB	−0.73 (−2.07 to 0.62)	0.294
		Time 3 months	−0.11 (−0.49 to 0.26)	0.554
		Time 6 months	0.37 (−0.01 to 0.75)	0.055
		Time 9 months	0.71 (0.34 to 1.09)	<0.001
		Age (years)	−0.07 (−0.21 to 0.08)	0.375
		Gender (male)	0.50 (−0.85 to 1.85)	0.468
		Malocclusion Class II	−0.36 (−1.69 to 0.97)	0.596
		Group SLB × Time 3 months	2.06 (1.52 to 2.59)	<0.001
		Group SLB × Time 6 months	2.26 (1.72 to 2.79)	<0.001
		Group SLB × Time 9 months	2.34 (1.81 to 2.88)	<0.001
	Passive extraction space closure	Intercept	4.62 (3.77 to 5.46)	<0.001
		Group (SLB vs. CB)	−0.15 (−0.48 to 0.18)	0.374
		Time: 3 months (T1)	−0.09 (−0.21 to 0.03)	0.161
		Time: 6 months (T2)	−0.17 (−0.29 to −0.05)	0.005
		Time: 9 months (T3)	−0.21 (−0.33 to −0.10)	<0.001
		Age (years)	−0.02 (−0.06 to 0.01)	0.217
		Gender (male)	0.11 (−0.22 to 0.43)	0.523
		Malocclusion Class II	−0.11 (−0.43 to 0.22)	0.520
		Group × Time 3 months	0.06 (−0.11 to 0.23)	0.508
		Group × Time 6 months	−0.07 (−0.24 to 0.10)	0.408
		Group × Time 9 months	−0.14 (−0.31 to 0.03)	0.099

Similarly, in the mandibular arch, SLBs achieved faster initial alignment. At 3 months, the additional reduction of 0.60 mm corresponded to an estimated time gain of 0.9 months (0.60 ÷ 0.70 ≈ 0.9 months) relative to CBs. At six and 9 months, the differences between bracket systems were smaller and not statistically significant. Overall, CBs required ~0.9 additional months to reach the same mandibular alignment as SLBs.

These results are in agreement with those reported by Jahanbin et al. [19], who observed significantly greater improvement in maxillary alignment during the first 4 months with Damon3 SLBs compared with MBT CBs. In their study, differences in mandibular alignment were largely restricted to the early phase of treatment and diminished over time. Similarly, Wahab et al. [20] found that CBs achieved faster alignment only during the first month, with no differences observed thereafter, implying that early treatment mechanics and ligation methods may play a more important role than the bracket design itself. Other trials also reported that less time is required to correct irregularity in SLB than in CBs [21]. Pandis et al. [18] found no overall difference in the duration required to resolve mandibular crowding between self‐ligating and conventional systems, although a relative advantage for SLBs was noted in patients with moderate levels of irregularity, suggesting that the initial severity of crowding may influence perceived treatment efficiency. In contrast, other investigations have not demonstrated a consistent benefit of SLBs. Scott et al. [14] reported no significant differences in mandibular alignment efficiency between Damon3 and CBs, concluding that the bracket type did not substantially influence the rate of alignment when confounding factors were adequately controlled. Evidence from systematic reviews further supports these observations. The quantitative review by Yang et al. [13] concluded that SLBs do not offer clear clinical superiority over CBs with respect to alignment efficiency or overall treatment duration, despite occasional short‐term advantages reported in individual trials. This suggests that early reductions in Little’s Irregularity Index associated with SLBs may not necessarily translate into meaningful long‐term clinical benefits.

From a biomechanical perspective, the early alignment advantage seen with SLBs can be attributed to reduced friction during the initial phase of treatment, allowing more efficient engagement of superelastic NiTi archwires. As treatment progresses and stiffer, larger‐dimension archwires are introduced, frictional differences between bracket systems become less relevant, which likely explains the progressive convergence of alignment outcomes observed at later stages [22].

In our study for intercanine width, no significant difference was observed between self‐ligating and CBs at 3 months in either the maxilla or mandible. For intercanine width, CBs demonstrated significantly greater expansion than SLBs throughout the study period. After adjustment for time, age, gender, and malocclusion, mixed‐effects regression analysis showed significant differences between the two bracket systems at 3, 6, and 9 months in both the maxillary and mandibular arches (all *p*  < 0.001). These findings indicate that CBs consistently produced greater increases in intercanine width than SLBs over time.

Previous studies, however, have reported mixed findings. Fleming et al. [15] in the UK observed a mean difference of −0.32 mm (95% CI: −1.15 to 0.51), indicating a slightly smaller increase with PSLB, although the difference was not statistically significant. Pandis et al. [18] in Greece reported a similar nonsignificant trend toward reduced expansion with PSLB (mean difference −0.50 mm; 95% CI: −1.06 to 0.06). In a multicenter randomized trial, Pandis et al. [16] reported a significantly smaller increase in intercanine width in patients treated with SLBs compared with CBs. Similarly, a meta‐analysis incorporating 17 clinical trials found a modest but statistically significant tendency toward greater intercanine expansion with CBs (mean difference −0.51 mm; 95% CI: −0.85 to −0.17) [17]. However, these findings should be interpreted with caution when compared with the present study. Most of the included studies, including those by Pandis et al., were conducted in nonextraction patients and primarily evaluated mandibular arch changes. In contrast, all participants in the present study underwent extraction treatment, which introduces different biomechanical considerations, including canine distalization into a wider portion of the dental arch and spontaneous dimensional changes associated with extraction space closure. These treatment‐related differences may partly explain the greater magnitude of intercanine width changes observed in our study compared with those reported in previous nonextraction investigations. Meanwhile, Scott et al. [14] in the USA, as well as several other randomized trials, observed nonsignificant differences between bracket types [3, 15]. Several factors may explain the variability in intercanine width reported across studies, including differences in age, gender distribution, severity of crowding, and malocclusion characteristics. Not all trials consistently reported or adequately controlled for these baseline variables, thereby limiting the direct comparability of outcomes. Additionally, individual variations in perioral musculature and functional forces may influence transverse dental changes during the alignment phase. Variations in treatment mechanics among studies, particularly regarding the use of adjunctive expansion appliances, may have further contributed to the observed heterogeneity in intercanine width changes. For example, the incorporation of expanders such as the quad helix in some conventional bracket protocols, as reported by Atik and Ciger [23], may have confounded the observed transverse changes and contributed to greater intercanine or intermolar expansion independent of bracket design.

The magnitude of the differences in our study is higher than those reported in previous trials, which largely involved nonextraction cases. In our cohort, all patients underwent extraction treatment, and canine distalization into a wider arch during passive space closure contributed to greater transverse expansion in both the maxilla and mandible. In the self‐ligating bracket (SLB) group, the mechanics resembled the effect of a Frankel appliance [12], where perioral soft tissue forces help retain canine expansion while allowing distalization without additional transverse expansion. This combination of passive space closure and natural perioral force restraint resulted in controlled canine movement yet still contributed to significant intercanine width changes. Consequently, the biomechanical effects of extraction, distalization, and SLB mechanics together explain why the observed differences between conventional and SLBs in our study are larger than those previously reported in nonextraction cases.

For intermolar width, SLBs demonstrated significantly greater transverse expansion than CBs throughout the observation period. Mixed‐effects regression analysis showed significant group‐by‐time interactions as early as 3 months, with mean differences of 2.34 mm in the maxilla and 2.06 mm in the mandible (both *p*  < 0.001). This difference increased at 6 months (maxilla: 2.54 mm; mandible: 2.26 mm; both *p*  < 0.001) and remained significant at 9 months (maxilla: 2.77 mm; mandible: 2.34 mm; both *p*  < 0.001). These findings indicate that SLBs achieved greater transverse intermolar expansion over time than CBs. The observed expansion is likely attributable to dentoalveolar tooth movement and minor buccal tipping rather than true skeletal widening. These results suggest that SLBs achieved modest but statistically significant transverse expansion over time, likely due to alignment‐related tooth movement and minor tipping rather than true skeletal widening. The greater increase in intermolar width observed with SLBs in the present study is best explained by dentoalveolar rather than skeletal mechanisms. Reduced friction in self‐ligating systems allows for more efficient expression of the preformed transverse archwire shape, facilitating lateral movement of posterior teeth during alignment and leveling. The use of broader archform wires further contributes to transverse development [23], primarily through buccal tipping and limited bodily movement of the molars within the alveolar bone rather than true skeletal expansion. Moreover, the absence of elastomeric ligatures may lessen perioral muscular constraints, permitting passive adaptation of the posterior segments to the archwire form over time. The early emergence of significant differences at 3 months, with progressive increases at six and 9 months, supports the notion that posterior expansion with SLBs occurs gradually through alignment‐related tooth movement and transverse archwire expression. These findings were further substantiated by mixed‐effects regression analysis, which confirmed significantly greater transverse expansion in both maxillary and mandibular intermolar widths with SLBs.

Atik et al. [23] conducted an RCT comparing SLBs and CBs in patients with Class I maxillary constriction. Although the increase in intermolar width was not statistically significant, it was greater in the CB group than in the SLB group. The difference between their findings and ours may be attributed to the use of a quad helix appliance for expansion in the CB group, while no expander was used in the SLB group. This suggests that SLBs alone are capable of producing intermolar expansion, as observed in our study. Another prospective randomized controlled trial comparing CBs and SLBs also found a significant difference in intermolar width, with greater expansion observed in the SLB group than in the CB group (*p* = 0.009) [15]. Similar other trials also shown that intermolar width is significantly large in patients receiving treatment with SLB (especially passive) than CB [4, 16, 18, 24].

Our findings showed that passive extraction space closure progressed similarly in both self‐ligating and CBs over a 9‐month period. Differences between groups at 3 and 6 months were not statistically significant, and the small difference observed at 9 months in the mandible was clinically negligible. Regression analysis confirmed that the bracket type did not influence space closure, with time being the main factor driving reduction. These results indicate that passive space closure is primarily determined by tooth alignment and archwire sequence rather than bracket design. Although SLBs reduce friction, this does not appear to provide a meaningful advantage during this phase of treatment, suggesting that both systems are equally effective for managing extraction spaces. Similar results were reported by previous studies [13, 25–28].

### 4.1. Clinical Implications

The observed reduction in alignment time of ~1.25 months, although statistically significant, represents a relatively small difference when considered in the context of overall orthodontic treatment duration and is therefore likely to have a limited clinical impact. While this difference may contribute to earlier improvement in alignment during the initial treatment phase, it is unlikely to influence the total length of treatment. In contrast, the ~2 mm increase in intermolar width may be regarded as clinically relevant, particularly in extraction cases where transverse arch control is important. However, the clinical significance of this finding should be interpreted with caution as the stability of the transverse changes and their underlying dentoalveolar or skeletal nature were not assessed. Further studies with longer follow‐up are required to determine the long‐term implications of these findings.

### 4.2. Limitations

This study has several limitations. First, incisor and molar inclinations were not measured, which may have influenced the interpretation of arch dimensional changes and space closure mechanics. Second, patients were not followed until completion of treatment, so possible differences in total treatment duration between the bracket systems could not be assessed. Third, treatment was carried out by more than one operator, which may have introduced some variation in clinical procedures. Although operator blinding was not possible, outcome assessment was performed by a blinded examiner to reduce measurement bias. Fourth, this was a single‐center study, which may limit the generalizability of the findings to other settings or populations. Fifth, the trial was registered retrospectively rather than prospectively, which should be considered when interpreting the overall methodological transparency of the study. Sixth, although age was included as a covariate in the statistical models, the study included adolescent patients who may have been at different stages of growth and development. Therefore, growth‐related changes in arch dimensions cannot be completely excluded as a potential confounding factor. Finally, cone‐beam computed tomography (CBCT) was not used to assess buccal bone support or dentoalveolar changes, so the biological basis and long‐term stability of the observed transverse changes could not be fully evaluated.

## 5. Conclusion

Within the limitations of this study, SLBs demonstrated faster early alignment, smaller increases in intercanine width, and greater intermolar expansion during the alignment phase compared with CBs. No significant difference was observed between SLBs and CBs with respect to passive extraction space closure. However, the magnitude of these differences was modest and did not support the overall clinical superiority of either appliance system. Therefore, bracket selection should continue to be based on clinical judgement, treatment objectives, and operator preference.

## Author Contributions

**Marium Jamil:** conceptualization, data curation, investigation, writing – review and editing. **Fareeha Bokhari**: conceptualization, data curation, investigation, writing – original draft, supervision. **Ayesha Hammad**: conceptualization, formal analysis, validation, writing – review and editing. **Hassaan Saeed**: conceptualization, formal analysis, validation, writing – review and editing. **Maliha Mumtaz**: conceptualization, methodology, investigation, writing – review and editing. **Muhammad Abdullah Kamran**: conceptualization, methodology, investigation, writing – review and editing. **Ali Alqerban**: conceptualization, methodology, writing – original draft, writing – review and editing, supervision. **Umar Hussain**: conceptualization, formal analysis, validation, writing – review and editing. **Ferdous Bukhary**: conceptualization, methodology, writing – original draft, writing – review and editing, supervision. **Asif Rehman**: conceptualization, data curation, formal analysis, methodology, investigation, writing – review and editing.

## Funding

This research did not receive any specific grant from funding agencies in the public, commercial, or not‐for‐profit sectors. The study was self‐funded by the authors.

## Disclosure

All authors reviewed the manuscript, approved the final version for publication, and agreed to be accountable for all aspects of the work. The corresponding author had full access to all study data and takes complete responsibility for the integrity of the data and the accuracy of the data analysis.

## Conflicts of Interest

The authors declare no conflicts of interest.

## Data Availability

The data supporting the findings of this study are available from the corresponding author upon reasonable request. Participant‐identifiable data cannot be shared due to privacy and confidentiality considerations.
